# The more we know, the more we have to discover: an exciting future for understanding cilia and ciliopathies

**DOI:** 10.1186/s13630-015-0014-0

**Published:** 2015-03-31

**Authors:** Alexandre Benmerah, Bénédicte Durand, Rachel H Giles, Tess Harris, Linda Kohl, Christine Laclef, Sigolène M Meilhac, Hannah M Mitchison, Lotte B Pedersen, Ronald Roepman, Peter Swoboda, Marius Ueffing, Philippe Bastin

**Affiliations:** INSERM U1163, Laboratoire des Maladies Rénales Héréditaires, 24 boulevard du Montparnasse, 75015 Paris, France; Institut Imagine, Université Paris Descartes-Sorbonne Paris Cité, 24 boulevard du Montparnasse, 75015 Paris, France; Centre de Génétique et de Physiologie Moléculaires et Cellulaires, CNRS UMR 5534, Université Claude Bernard Lyon 1, 16 rue Dubois, Villeurbanne, Lyon, F69622 France; Department of Nephrology, University Medical Centre Utrecht, 100 Heidelberglaan, Utrecht, 3584CX The Netherlands; The Ciliopathy Alliance, 91 Royal College St, NW1 0SE, London; UMR7245 CNRS/MNHN, Muséum National d’Histoire Naturelle, 57 rue Cuvier, 75005 Paris, France; Developmental Biology Laboratory UMR7622, UPMC Univ Paris 06, Sorbonne Université, 9 Quai Saint Bernard, F-75005 Paris, France; Developmental Biology Laboratory UMR7622, CNRS, Institut de Biologie Paris Seine (IBPS), 9 Quai Saint Bernard, F-75005 Paris, France; INSERM, ERL1156, 9 Quai Saint Bernard, F-75005 Paris, France; Department of Developmental and Stem Cell Biology, Institut Pasteur, 25 rue du docteur Roux, 75015 Paris, France; CNRS URA2578, 25 rue du docteur Roux, 75015 Paris, France; Genetics and Genomic Medicine, Institute of Child Health, University College London, 30 Guilford Street, London, WC1N 1EH UK; Department of Biology, University of Copenhagen, Universitetsparken 13, 2100 Copenhagen, OE Denmark; Department of Human Genetics, Radboud University Medical Center, Geert Grooteplein 25, P.O. Box 9101, 6525 Nijmegen, GA The Netherlands; Department of Biosciences and Nutrition, Karolinska Institute, Hälsovägen 7, S-141 83 Huddinge, Sweden; Institute for Ophthalmic Research, University of Tübingen, PO 2669, D-72016 Tübingen, Germany; Research Unit of Protein Science, Helmholtz Zentrum München, German Research Center for Environmental Health, D-85758 Neuherberg, Germany; Trypanosome Cell Biology Unit, Institut Pasteur and INSERM U1201, 25 rue du Docteur Roux, 75015 Paris, France

**Keywords:** Cilia, Flagella, Basal bodies, Centrioles, Ciliopathies, Intraflagellar, Transport, Signalling, Hedgehog, Development

## Abstract

The Cilia 2014 conference was organised by four European networks: the Ciliopathy Alliance, the Groupement de Recherche CIL, the Nordic Cilia and Centrosome Network and the EU FP7 programme SYSCILIA. More than 400 delegates from 27 countries gathered at the Institut Pasteur conference centre in Paris, including 30 patients and patient representatives. The meeting offered a unique opportunity for exchange between different scientific and medical communities. Major highlights included new discoveries about the roles of motile and immotile cilia during development and homeostasis, the mechanism of cilium construction, as well as progress in diagnosis and possible treatment of ciliopathies. The contributions to the cilia field of flagellated infectious eukaryotes and of systems biology were also presented.

**Electronic supplementary material:**

The online version of this article (doi:10.1186/s13630-015-0014-0) contains supplementary material, which is available to authorized users.

## Introduction

In 2012, the Ciliopathy Alliance organised in London what was probably the first international meeting solely dedicated to cilia and ciliopathies in Europe. Following the success of the Cilia 2012 conference (http://www.ciliajournal.com/supplements/1/S1), four European networks worked hand in hand to organise the Cilia 2014 conference on the campus of the Institut Pasteur in Paris. These were the Ciliopathy Alliance (http://www.ciliopathyalliance.org/), the Groupement de Recherche CIL (http://gdr-cil.snv.jussieu.fr/), the Nordic Cilia and Centrosome Network (http://nordiccilia.org/) and the EU FP7 programme SYSCILIA (http://syscilia.org/). With 411 delegates from 27 countries encompassing 5 continents, we can say that Cilia 2014 was a huge success. The meeting also attracted 30 patients and patient representatives and offered the unique opportunity for exchange between different communities.

The meeting was opened by Philippe Bastin (Institut Pasteur, Paris), the chair of the organising committee, who recalled the sentence of famous French scientist Louis Pasteur ‘There is no such thing as applied research and fundamental research. There is only research and its applications, united one to the other, as the fruit is linked to the branch that carried it’. More than a century later, this is incredibly true for the primary cilium. This organelle was long considered to be vestigial and to serve no function [1]. In 2000, Greg Pazour and co-workers identified the sequence of the *IFT88* gene in the green alga *Chlamydomonas* during a purely fundamental research project. This was the follow-up of the identification of intraflagellar transport (IFT) and its protein components in *Chlamydomonas* [2,3]. They next discovered *IFT88* corresponded to *Tg737*, a gene mutated in a mouse model for polycystic kidney disease, and pushed on by investigating mouse kidneys with the presence of cilia at the surface of epithelial cells in normal tubules. These were severely stunted in the *Tg737* mouse kidneys, hence providing the first connection between primary cilia and a genetic disease [4]. The gene is conserved in *Caenorhabditis elegans* and its protein product turned out to be essential for IFT, cilium construction and proper polycystin localisation [5-7]. These seminal discoveries prompted further research, and the field blossomed rapidly. Work using multiple human genetic analyses as well as a wide range of model organisms has resulted in spectacular progress in diagnosis of ciliopathies, fittingly within the heritage of Louis Pasteur’s citation.

### Motile cilia, laterality and primary ciliary dyskinesia

**Figure 1:**
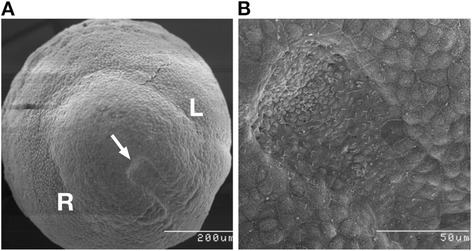
**Scanning electron micrographs of an early mouse embryo, with three somite pairs, viewed from the posterior. (A)** The node (white arrow) is clearly visible as a depression with a rounded posterior that continues forward opening out into the notochord. The cells surrounding the top of the node are often called the crown cells, and these have been implicated in responding to leftward nodal flow. Left (L) and right (R) sides are indicated. **(B)** A higher magnification of the node shown in panel (A). At this magnification, the presence of a single cilium on each of the cells within the pit of the node is visible. These cilia beat in a circular manner, but due to a posterior tilt, they drive a leftward flow of fluid in the node. It is this leftward nodal flow that normally establishes the direction of the left-right axis (credit: Dominic Norris).

The plenary speaker Martina Brueckner (Yale University, USA) opened the scientific part of the meeting with an introduction to the central role of cilia in laterality and heart development. Heterotaxies affect 1 in 20,000 births, with 90% of heterotaxy patients exhibiting lethal congenital heart defects. The process of left-right determination is highly conserved through evolution. The involvement of cilia in this process was first predicted by Afzelius in 1976 [8]. Subsequently, it was revealed that the embryonic node (Figure 1) is a ciliated left-right organiser (LRO) that contains motile cilia that drive a leftward flow of fluid; the surrounding crown cells have primarily non-motile sensory cilia [9]. The ciliary ‘nodal flow’ theory proposes that cilia-based fluid flow in the early embryonic node establishes ‘leftness’ either due to (1) a morphogen becoming asymmetrically distributed in response to flow or (2) mechanosensation of flow by non-motile cilia on the crown cells (also known as the two-cilia hypothesis). One possible role for cilia in this process is that they represent calcium signalling organelles that can respond to fluid flow [10]. To test this hypothesis, cilia-targeted (ARL13b-linked) calcium indicators were created to image intraciliary calcium signalling. This identified novel intraciliary calcium oscillations (ICO) inside the cilium during development. These are regulated in both a motility- and polycystin-2 calcium channel-dependent manner. ICO are the first sign of asymmetry and occur when motile cilia are first seen at the node: ARL13b-targeted removal of calcium from cilia interferes with embryonic laterality. Leftward flow triggers ICO and subsequent left-right asymmetric gene expression that is upstream of asymmetric organ development. Martina Brueckner speculated that cilia provide a unique environment for these functions, being an isolated calcium compartment with a high membrane to cytoplasm ratio, giving the ability for quick calcium responses.

Julien Vermot (IGBMC, Strasbourg, France) also addressed this still unsolved question of how fluid dynamics created by beating cilia can mediate left-right asymmetry during vertebrate development. Using live imaging of motile cilia in the zebrafish Kupffer’s vesicle, combined with 3D reconstruction and modelling of cilia parameters, he challenged the existing hypotheses discussed above: mechano-sensation by sensory cilia in the periphery of Kupffer’s vesicle (two cilia hypothesis) or asymmetric morphogen distribution. He concluded that the current biological assumptions fail to fully explain symmetry breaking with the recorded physical parameters of cilia induced flow, bringing novel insights into the fascinating field of left-right asymmetry specification in animals. An award-winning poster from the same group presented by Rita Rua Ferreira showed an innovative approach to study cilia positioning in the establishment of the left-right axis in zebrafish. Using an Arl13b-eGFP transgenic line displaying fluorescent cilia, she was able to generate a 3D topographic map of the wild-type zebrafish left-right organiser, showing that cilia are oriented towards the dorsal pole of the left-right organiser at a 60° angle on the cell surface. She also showed that myosin II is important in the positioning of cilia and the production of directional flow, but not in the orientation of cilia, indicating the involvement of other orientation cues.

Two talks in the development session highlighted the different mechanisms underlying cilia motility in nodal and tracheal cells, carrying respectively a single 9 + 0 cilium and hundreds of 9 + 2 cilia. Jennifer Keynton (MRC Harwell, UK) presented a new mutant mouse model for primary ciliary dyskinesia (PCD) carrying a mutation in the axonemal dynein heavy chain (*Dnah11*), called *lrm5*, which shows normal ciliary beat frequency in the trachea, but accelerated and abnormal cilia motion in the node. Kyosuke Shinohara (Osaka University, Ibaraki, Japan) demonstrated that taxol treatment, which stabilises microtubules, alters nodal cilia motility as well as the internal organisation of the nine microtubule doublets. The same treatment does not affect cilia movement in tracheal cells, unless they miss the radial spoke protein RSPH4a. Thus, the regular ninefold symmetry of microtubule doublets is essential for cilia motility in both cell types, but the exact role of radial spoke components in ciliary motility and structure remains elusive.

Cecilia Lo (University of Pittsburgh, USA) presented a powerful, unbiased forward genetic screen for cardiac defects in mouse, using non-invasive foetal echocardiography. This screen identified at least 38 novel genes not previously implicated in the aetiology of congenital heart defects and uncovered genes involved in cellular trafficking, cell signalling and many genes that affect different steps of motile cilia biogenesis or function. The full collection is available at (http://www.jax.org/search/Main.jsp?qt=bench+to+bassinet&sg=0).

In addition to the incredible value of this collection for genetic understanding of cardiac defects, this screen revealed novel functional understandings for genes involved in PCD.

Another approach to identify candidate PCD genes was presented by Girish Mali (University of Edinburgh, UK). He reported a comprehensive update on work in *Drosophila* using the RFX-FOX transcriptional code to identify novel candidate genes important for the assembly and function of motile cilia [11]. Of note, human orthologues of two of the identified fly genes have recently been shown to be involved in PCD [12]. The authors performed endogenous-immuno-precipitation mass spectrometry (IP/MS) to define the protein interactomes for some of the newly identified gene products and found - not surprisingly - complex interaction patterns, including with ciliary motility and vesicular trafficking proteins. Fittingly, mice mutant for one of the identified proteins showed ciliary defects evident in sperm flagella motility. Thus, contrary to what many might have thought, *Drosophila* is proving a useful model for studying human PCD.

Motile cilia are ‘one per cell’ in the embryonic node as well as in spermatozoa. Vera Jansen (Caesar Research Centre, Bonn) presented an award-winning poster reporting a very powerful and interesting approach to study cAMP in spermatozoa; she generated and analysed a transgenic mouse expressing a photoactivatable adenyl cyclase in sperm. This model allows precise spatio-temporal control of the intracellular cAMP concentration, facilitating the dissection of cAMP-dependent pathways in the sperm flagellum.

Multiple motile cilia are present in epithelial cells of the respiratory tract, the brain ventricles and the oviduct. Multi-ciliated cells are required to perform efficient mucociliary clearance and/or fluid transport. Four presentations brought novel and exciting insights into the mechanisms by which centriole amplification occurs in multi-ciliated cells and the molecular cascades regulating this process. Alice Meunier (Ecole Normale Supérieure, Paris, France) described novel data on how the daughter and mother centrioles adopt specific fates to produce hundreds of centrioles for multiple cilia formation. By combining super-resolution and electron microscopy, she demonstrated that the daughter centriole leads to successive rounds of deuterosome formation and procentriole nucleation, whereas the mother centriole joins in the dance at the last round of centriole amplification by directly nucleating novel centrioles [13]. Julia Wallmeier (University of Münster, Germany) presented how the upstream factor MCIDAS regulates not only deuterosome formation by regulating *CCNO* (cyclin O) or *CCDC78* genes but also *FOXJ1* induction, and hence, genes required for motility [14,15]. Sebastian Arnold (University of Freiburg, Germany) presented data showing that genetic deletion of *Ccno* resulted in impaired multi-ciliated cell generation and growth. This impairment is caused by compromised deuterosome affecting centrosome amplification. *Ccno*-deficient mice display gross hydrocephalus and mucociliary clearance defects and severe airway diseases as do *CCNO* mutations in humans. *Ccno*-null mice offer a new way to study centriole amplification by deuterosome. Finally, Laure-Emmanuelle Zaragosi (Institut de Pharmacologie Moléculaire, Sophia-Antipolis, France) described the function of *CDC20B*, another gene within a specialised locus that encompasses at least four members described to be required for motile ciliogenesis programme: *miR-449, CDC20B, MCIDAS* and *CCNO*. CDC20B represents a novel protein involved in deuterosome-mediated centriole amplification and basal body docking in multi-ciliated cells.

### Multiple roles for cilia during development

**Figure 2:**
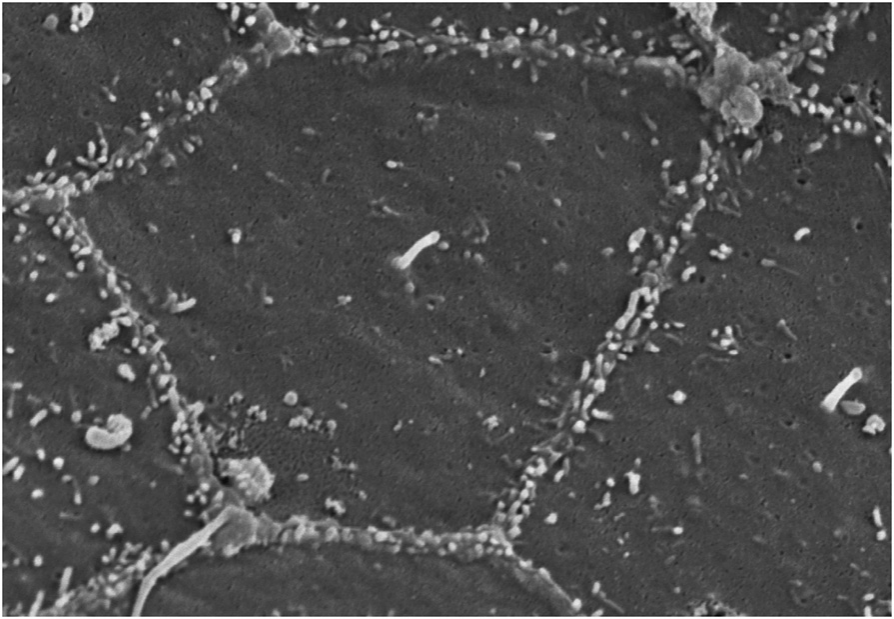
**The primary cilium.** Scanning electron micrograph of the buccopharyngeal epithelium of E9.5 mouse embryo, showing a central primary cilium emerging from the apical surface surrounded by microvilli (credit: Christine Laclef).

During embryonic development inductive signals are exchanged between cells, driving morphogenesis and specifying cell fate in a precise spatio-temporal manner. We have already seen how motile cilia contribute to establishing left-right asymmetry. Further presentations at the meeting illustrated how primary cilia (Figure 2), as sensory organelles, play major roles in other aspects of development. One question addressed was how cilia are specialised in different cell types, to sense a specific signal. Transcription factors, such as Rfx and Forkhead box Fd3F, have been identified as upstream regulators of a gene network underlying both ciliogenesis and acquisition of cilium specialisation. Andrew Jarman (University of Edinburgh, UK) showed that in the fly, in which only sensory neurons and sperm have primary cilia, *Rfx* is required for ciliogenesis, whereas *Rfx* and *Fd3F* cooperate to specify the motility apparatus of the cilium in chordotonal neurons [16]. However, ectopic expression of these factors is not sufficient to induce a primary cilium in non-ciliated cells or to drive motility of non-motile primary cilia. Targets of *Fd3F* include genes involved in the assembly of axonemal dyneins, and analysing such targets led to the identification of two new dynein assembly factors, *Zmynd10* [12] and *Heatr2* [11].

**Figure 3:**
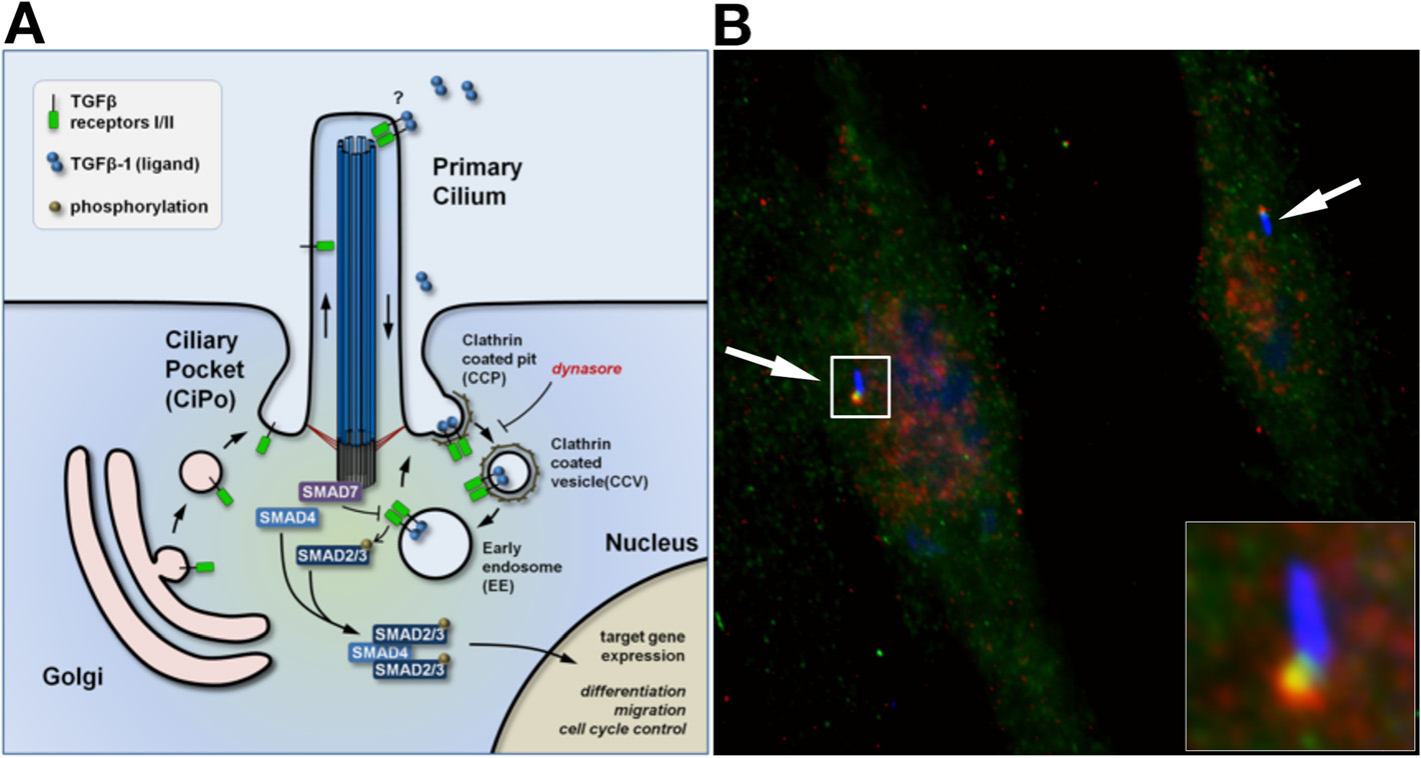
**Example of signalling at the primary cilium. (A)** TGFβ/BMP signalling at the primary cilium. **(B)** TGFβ-RI (green) and P-smad2/3 (red) are recruited at the base of the primary cilium (blue) in TGFβ1-stimulated human foreskin fibroblasts (credit: [17]).

Among the signals transduced in the ciliary region, Søren Christensen (University of Copenhagen, Denmark) provided evidence that signalling of the transforming growth factor β (TGFβ)/bone morphogenetic protein (BMP) super-family involves primary cilia. He showed that activation of SMAD2/3 and SMAD1/5 transcription factors upon TGFβ/BMP stimulation is regulated partly by the trafficking of receptors within the primary cilium and partly by the internalization of the receptors by clathrin-dependent endocytosis at the ciliary pocket [17]. His observations also suggest that TGFβ/BMP signalling is modulated by a negative feedback loop at the cilium, mediated by SMAD7 and the ubiquitin ligase SMURF1, to control the balancing of stem cell differentiation into either cardiomyocytes or neuronal cells (Figure 3). Jantje Gerdes (Helmholtz Zentrum München, Germany) presented results on insulin signalling, which is required for its own secretion by pancreatic β cells. Insulin secretion requires localisation of the insulin receptor in the primary cilium. This provides for the first time insight into the origin of the higher risk of type 2 diabetes in patients with ciliopathies [18]. In human mesenchymal stem cells, Melis Dalbay (Queen Mary University of London, UK) reported that insulin destabilises primary cilia. Destabilisation of the length of primary cilia, with siRNA knocking down *IFT88,* decreased trafficking of insulin-like growth factor receptor 1β and thereby impaired adipogenic differentiation.

The importance of cilia in vertebrate development was first revealed in genetic experiments showing that primary cilia are required for survival and morphogenesis of the mouse embryo [19]. Phenotypic, genetic and biochemical analyses then showed that embryonic phenotypes of the ciliary mutants were caused by disruption of the Hedgehog (Hh) signal transduction pathway, which is essential for the development and patterning of many organs. In particular, the primary cilium is fundamentally important in transducing the canonical Hh pathway in the neural tube. Tamara Caspary (Emory University, Atlanta, USA) addressed the role of the ciliary protein ARL13b in cell migration and axon guidance. She showed that Sonic Hedgehog (SHH)-dependent migration is impaired in *Arl13b*-null fibroblasts and is not rescued by non-ciliary or mutated forms of Arl13b described in Joubert patients. In addition, she provided evidence for ARL13b function, likely mediated by Hh signalling, in axon guidance in commissural neurons of the posterior neural tube.

Until recently, it was the prevailing view that cilia-based Hh signalling is restricted to vertebrates. Mattias Alenius and his colleagues (Linköping University, Linköping, Sweden) have now shown that Hh signalling also takes place in the cilia of invertebrates, more precisely in the single sensory cilium of olfactory neurons of the *Drosophila* antenna [20]. They show that ‘all the relevant parts’ of the Hh signalling pathway, for example, Smo, localise to fly cilia as they should and that proper localisation is abrogated upon patched mutation. Furthermore, functional relevance of the Hh signalling pathway in cilia, including proper signal transduction output, was demonstrated in behavioural assays such as odorant sensation (‘smell’), again using thorough analysis of mutants. This work sheds new light on the co-evolution between components of the Hh signalling pathway and the function of sensory cilia.

### Ciliopathies: when cilia go wrong

The opening session of the conference introduced the medical importance of the growing class of inherited diseases arising from mutations in cilia-associated proteins that play roles in the structure and function of cilia, centrosomes and flagella. ‘Ciliopathy’ diseases are both pleiotropic and genetically heterogeneous conditions with symptoms affecting multiple systems, reflecting the ubiquitous nature of sensory and motile cilia. They share not only clinical features, predominantly cystic kidney disease, but also retinal, respiratory, skeletal, hepatic and neurological defects in addition to obesity, laterality defects and polydactyly. An overview from Phil Beales (University College London, UK) gave a flavour of the range of sensory ciliopathies by describing their clinical heterogeneity affecting many different organ systems, as well as some of their unifying characteristics such as renal cystic disease. In Bardet-Biedl syndrome (BBS), important genotype-phenotype correlations now allow some prognostic predictions of disease outcomes for certain mutations. In ciliopathies, the delineation of specific mutations can provide a stratified patient population suitable for differently genetically targeted therapies. This is exemplified in BBS where particular mutations are being tested for their amenability, for example, to non-sense read-through and antisense splicing therapeutic rescue techniques. Genetic definition of Jeune syndrome is nearly complete, with isolation of several new disease genes including unexpected novel ones such as *CEP120* [21]. Medically relevant genotype-phenotype correlations have also become apparent: mutations in genes encoding components of the retrograde IFT dynein-2 motor, and some IFT-B particle (for example, *IFT80*) components cause mainly skeletal defects, while IFT-A and some IFT-B (for example, *IFT172*) defects have extra-skeletal complications, such as severe renal disease in the case of *IFT140* mutations [22,23]. As with other ciliopathies, there is phenotypic variability. Miriam Schmidts (Radboud University Nijmegen, The Netherlands) reported on the results of work undertaken while a student at UCL relating to sequencing DNA from over 300 individuals with Jeune syndrome, which identified 3 families with mutations in a new cytoplasmic IFT dynein-2 light chain, *TCTEX1D2* (work undertaken when she was at UCL). These families had clinically unaffected members carrying mutations, suggesting incomplete penetrance. Proteomics analysis confirmed that TCTEX1D2 is part of the IFT dynein-2 complex. Impairment of retrograde IFT seemed milder compared with other dynein-2 defects. Indeed, loss of TCTEX1D2 in the human cells and *Chlamydomonas* resulted in no apparent change in ciliary structure.

Focussing on human diseases connected to defects in primary cilia function, Sophie Saunier (Imagine Institute, Necker Hospital, France) reported progress with genetic analysis of a large (>800 families) cohort of nephronophthisis (NPHP) patients. NPHP-associated ciliopathies comprise a group of autosomal recessive diseases including either isolated NPHP or Senior-Løken, Joubert, Jeune and Meckel-Gruber syndromes, presenting fibrotic and cystic kidneys with retinal degeneration and skeletal and cerebellar/neural tube malformation. At the cellular level, defects are observed in ciliogenesis, ciliary membrane composition, IFT localisation and ciliary signalling. A total of 271 NPHP cases (88 isolated NPHP and 183 syndromic forms) were subject to high-throughput ciliome sequencing using a targeted gene panel created at the Imagine Institute. Causal mutations in 20 new genes were identified, including *CEP83* [24] underlying infantile NPHP, which, like the two other ciliopathy-associated genes *SCLT1* and *CEP164*, encodes a centriole distal appendage component. Mutations in several components of the intraflagellar transport B particle were also detected. Further work on IFT54 (*TRAF3IP1*) showed that disease mutations affect its intraciliary localisation as well as ciliary membrane composition causing a defect in cAMP/PKA ciliary signalling. They also affect the binding of IFT54 to microtubules and to MAP4, a microtubule-associated protein involved in ciliogenesis (see also presentation by G. Pereira below). As a consequence, MAP4 is missing from cilia in IFT54-mutated patient cells and its recruitment to cytoplasmic microtubules is increased causing microtubule stabilization and hyper acetylation. Valentina Grampa (same group) won a best poster award reporting her work identifying several recessive *NEK8* mutations in patients with severe ciliopathies. In patient fibroblasts, the localisation of NEK8 was shifted to the Golgi, and its ciliary partner ANKS6/NPHP16 was also mis-localised. In zebrafish, injection of *nek8* morpholinos led to ciliopathy-related phenotypes that could be rescued by wild-type *nek8*, but not by the mutated forms. This work showed that Nek8 is essential for ciliary and non-ciliary processes (cell cycle-related) and mutations in this protein result in multi-systemic defects. Interactions between cilia and the Golgi apparatus have also been observed by Dylan Bergen (University of Bristol, UK) who won an award for best poster presentation. He used a morpholino knockdown approach to show that in the absence of the Golgi matrix protein giantin, zebrafish display severe abnormalities both in extra-cellular matrix deposition, resulting in craniofacial defects, and in cilia function, such as randomisation of the heart position. His work implies a dual role for giantin, both in intracellular matrix secretion and in cilium function.

Gisela Slaats (University Medical Centre Utrecht, The Netherlands) further described CEP164 as having an important role in regulating cilia and the DNA damage response in NPHP. Primary ciliated cells isolated from urine of a NPHP patient showed enhanced DNA damage and nuclear CEP164, and a quicker cell cycle but delayed S phase was demonstrated after *CEP164* knockdown in RPE-Fucci (fluorescent, ubiquitination-based cell cycle indicator) cells, although no enhanced proliferation was seen. Apoptosis and epithelial-to-mesenchymal transition also increase after siRNA-mediated CEP164 depletion. Increased DNA damage could cause apoptosis, which is known to contribute to initiation of renal cyst formation. After siRNA knockdown of CEP164, expression of *Snail* is increased, characterising a pro-kidney fibrosis mesenchymal transition. Thus, loss of CEP164 affects ciliary function, along with DNA damage response, apoptosis, creation of a pro-fibrotic environment and proliferation, via pathways as yet to be discovered, which can all contribute to the NPHP renal phenotype [25]. Jean Piero Margaria (University of Turin, Italy) highlighted the role of a PI3K isoform, PI3K-C2α encoded by the *Pik3c2a* gene, in the process of renal cyst formation. PI3K-C2α localises to the ciliary base, and *Pik3c2a*-silenced IMCD3 cells and kidney tubules of *Pik3c2a*^+/−^ mice show ciliary defects with involvement of PI3K-C2α in the RAB8-dependent trafficking of polycystin-2 towards the primary cilium. These defects can be rescued by transfection of constitutively active RAB8. An over-activation of proliferative pathways regulated by ciliary polycystins is seen in *Pik3c2a*^+/−^ cells, with increased mTOR and MAPK signalling due to loss of their inhibition via polycystin calcium signalling. Increased proliferative signals predispose cells to cyst development.

In Joubert syndrome patients, the most common genetic abnormalities are mutations in *CEP290* (*NPHP6*). This gene is also mutated in several other ciliopathies [26]. Rachel Giles (University Medical Centre Utrecht, The Netherlands) showed that defective Hh signalling is implicated in NPHP and that an Hh agonist offers potential therapeutic opportunities. Cilia in the *Cep290*^*−/−*^ mouse model are very short and scarce; cystic kidneys form and progress from birth, as in humans with Joubert syndrome. Initially, abnormal Wnt signalling was expected but defective Hh signalling was instead observed, and this data was reproduced using a 3D cell culture model, generating spheroids from primary renal cells from mice. By treating the cells with a Hh agonist, the spheroid formation defects observed in cells from the *Cep290* mice were rescued. This approach was successfully replicated in human renal cells from a Joubert syndrome patient, suggesting that it may be possible to intervene at an early stage in the formation of kidney cysts [27]. Oliver Blacque (University College Dublin, Ireland) presented a new transition zone (TZ) protein mutated in Joubert syndrome: TMEM107. *C. elegans* TMEM107 regulates the integrity of the TZ diffusion barrier and associates with anchored ring-like subdomains of the TZ membrane.

Nine Knoers (University Medical Centre Utrecht, The Netherlands) examined clinical perspectives of reaching a diagnosis in a family with a ciliopathy. Whole exome sequencing is an efficient process valuable for gene discovery, understanding mutational burden, and eventually genotype-phenotype analysis, but from the point of view of the patients and their parents, this can be a long and possibly disappointing process [28].

Now that significant progress has been achieved in terms of diagnosis, the next challenge is how to translate these new discoveries towards therapies. Daniel Chung (University of Pennsylvania, Philadelphia, USA) reported successful adeno-associated virus (AAV)-mediated gene therapy clinical trials for retinal dystrophies caused by mutations in the *RPE65* gene, providing evidence that AAV technology could potentially be used to treat the auditory, ocular and renal effects in ciliopathies such as BBS and Usher syndromes and polycystic kidney diseases. The safety and efficacy of AAV RPE65 was demonstrated initially in blind mutant Briard dogs and then in 12 human subjects with retinal degeneration (the youngest being 8 years) in phase I/II trials [29]. A phase III open-label trial has since commenced on 24 subjects. This transgenic method and technology was next used on embryonic and adult mice that were given intraotocyst, ocular and renal injections of a CMV-promoted *EGFR* and/or a luciferase transgene. The mice tolerated the treatment, locally and systemically. In the otocyst, the AAV vectors transduced cochlear hair cells. Subretinal injection in adult mice transduced high levels of the transgene in ciliated photoreceptor cells. Transgene expression was also observed in collecting ducts of the kidney tubule cells.

Jane Sowden (Institute of Child Health, University College London, UK) demonstrated impressive and successful transplantation of 3D embryonic stem cell-derived retinal differentiation cultures into mouse models of retinal disease, leading to restored visual acuity [30]. This approach holds great promises for patients. Using a patented pharmacological approach called GV-Ret, to block caspase-12, Xiangxiang Yu (University of Strasbourg, France) discussed her work suppressing retinal degeneration in retinal explant models of BBS.

### Systems biology of cilia and flagella

Systems biology is a highly interdisciplinary road to biological discovery. It has regained momentum in recent years as a consequence of the dramatic increase in the rate of data accumulation by molecular biology and its offspring, functional and structural genomics. The broad aims of systems biology are to understand, model and predict the behaviours of biological networks of interaction and in particular their dynamic aspects and spatial developments. Small, relatively isolated systems are often targeted by systems biology approaches under the assumption that a limited set of molecules and interactions will be more tractable for modelling systems and that the system is not overly influenced by other systems, thereby over-complicating the models. Cilia are therefore ideal organelles for systems biology as they tick all of these boxes. Early proteomic studies suggest a discrete repertoire of about 1,000 proteins within the organelle (that is, <5% of the human proteome) that largely are still in need of organisation into networks and pathways. A broader understanding of the ciliary system could also provide explanations of how system failure could lead to the large genetic heterogeneity and clinical diversity of ciliopathies [31].

Martijn Huynen (Radboud University Medical Center, Nijmegen, The Netherlands) demonstrated how a method called ‘naïve Bayesian data integration’ could be used to generate a ranking of the likelihood of each human gene to be involved in a ciliary function. The integrated data ranged from weighted (co-)expression data, the sharing of conserved transcription factor binding sites, and the evolutionary distribution of genes among ciliated and non-ciliated species to physical interactions with known ciliary proteins, as determined by the SYSCILIA consortium. The resulting Bayesian ranking can also be used to predict the potential candidacy of a gene to be mutated in a ciliopathy, which is currently important to evaluate exome sequencing data of patients with (suspected) ciliopathies.

Systematic perturbation of a system is a powerful way to identify submodules that are essential for system function. Katarzyna Szymanska (University of Leeds, UK) showed the results of a complete systemic perturbation of the ciliary system, a genome-wide siRNA screen for defects in biogenesis and/or maintenance of the primary cilium. Stringent filtering and validation procedures to overcome experimental variation yielded a gene set that robustly affected ciliogenesis upon downregulation. The screen allowed the identification of not only anticipated important players but also perhaps surprising functional gene classes such as those encoding proteins involved in the ubiquitin-proteasome system, G-protein-coupled receptors and pre-mRNA processing factors. Screen hits also yielded new candidate ciliopathy disease genes, demonstrating one of the many utilities of the screen’s output for researchers in the field.

Marius Ueffing (University of Tübingen and Helmholtz Center München, Germany) provided the audience with a ‘Google earth’-like systems view of the basics of vision. He explained how a systematic analysis using quantitative, proteome-centric biochemistry, structural biology-based modelling and extensive *in silico* network analysis could lead to a new and improved understanding of the functional, multi-scale complexity of the photoreceptor sensory cilia and outer segments. Overall, these studies identified specific ciliopathy disease susceptibility within the core visual pathway and ciliary transport but a relative robustness of the other functional modules of the photosensory cilium. This session showed that systems biology approaches allow insights into common and heterogeneous properties of the ciliary system that provide a versatile, broad, relational view of ciliary function and dysfunction in ciliopathies.

Functional diversity of cilia provides a rationale for the heterogeneity of ciliopathies. Monica Bettencourt-Dias (Instituto Gulbenkian de Ciencia, Portugal) has mapped the diverse functions of the four classes of cilia of the fruit fly with great spatial resolution, using EM and super-resolution microscopy. Current concepts relate the functional diversity of cilia to diversity of the axoneme and its surrounding (membrane) compartments, harbouring the core of the ciliary receptors and signalling components. She showed that also, and maybe even more prominently, the base of the cilium (basal body and transition zone region) that is evolutionary highly conserved in protein content shows great diversity in number, length, ultrastructure and connection to other cellular structures. This provides an additional layer of complexity to understanding and predicting the tissue-specific phenotypes observed in ciliopathies.

### Cilia and flagella in infectious eukaryotes

Cilia and flagella are conserved in most eukaryotes, including in protists that are responsible for severe human or animal diseases, where they often play essential functions. Because of their early evolutionary divergence and because of the adaptations due to parasitism, protists exacerbated some cellular features that make them attractive models for cell biology in general. For example, dynein was first identified in ciliates [32] whereas the GPI anchor was first discovered in African trypanosomes [33].

Kent Hill (University of California Los Angeles, USA) presented a global overview of the biological roles of the flagellum of *Trypanosoma brucei*, the parasite responsible for sleeping sickness. This organism possesses a single flagellum that is essential for motility, morphogenesis, cell division and adhesion to some tissues in the tsetse fly [34]. It also emerged as a potent model to study cilia and flagella thanks to its amenity to transfection, RNAi, endogenous tagging and imaging. He reported analysis of the BBSome, showing conservation of the core complex in very distant eukaryotes. Stunning immunogold images support a role for the *T. brucei* BBSome at membranes while BBSome disruption indicates a role in infection, bridging cell biology and parasitology. Additionally, he reported that flagellar proteins controlling cAMP homeostasis are important for cell-cell interactions in trypanosomes, supporting a signalling role for the flagellum in these pathogens.

The flagellar apparatus of trypanosomes is characterised by amazing supplementary structures including the flagellar connector, a trilaminar structure of 400 nm in length found at the tip of the new flagellum during its synthesis that glides along the old flagellum [35]. The characterisation of this small structure has been hampered by its strong association with axonemes. Vladimir Varga (University of Oxford, UK) developed a novel immunoprecipitation-based approach that allows identification of protein constituents of discrete structures such as the flagellar tip and flagella connector. Remarkably, two components are molecular motors of the kinesin family.

*Leishmania* parasites are distant cousins to trypanosomes that are able to invade macrophages where they differentiate from the promastigote stage, that possesses a long 9 + 2 flagellum, to the amastigote stage, where a short immotile 9 + 0 flagellum is present, barely emerging from the flagellar pocket. Richard Wheeler (University of Oxford, UK) investigated the ultrastructure of flagella during differentiation from the promastigote to the amastigote stage both in axenic culture and during infection of macrophages. The results indicate that two pathways exist as follows: (1) an asymmetric division initiated with formation of a short 9 + 0 flagellum, a feature recently reported for the formation of different types of flagella in trypanosomes [36-38] or (2) the existing flagellum shortens and restructures via removal of components at the distal tip. This reveals that axonemes have flexibility in their structure and can convert from 9 + 2 to 9 + 0 and also supports the hypothesis that the immature basal body is not pre-programmed to assemble a given type of axoneme. The actual function of the short flagellum in amastigotes remains enigmatic. The award-winning poster of Tiffanie Fowlkes-Comninellis brought some intriguing insights. She studied the effect of the absence of IFT140, a well-characterised intraflagellar transport protein, in *Leishmania donovani*. Using a plasmid segregation knockout approach, she showed that parasites lacking IFT140 do not appear to possess a flagellum yet still grow normally.

An extreme case of flagellar specialisation is encountered during the formation of the male gamete in *Plasmodium*, the parasite responsible for malaria. The axoneme is assembled in the cytoplasm at the incredible rate of more than 1 μm/min, does not require IFT and gets wrapped up by a membrane when it is expelled from the cell by ‘exflagellation’ [39]. Sara Marques (Imperial College London) revealed that the construction relies on the presence of SAS6 [40] but more surprisingly of the radial spoke protein RSP9. It is proposed that RSP9 acquired supplementary functions in *Plasmodium*, perhaps to increase stability of the rapidly growing axonemes. This is supported by the presence of unusual extensions in the *Plasmodium* RSP9 sequence.

### Mechanisms for construction of cilia and flagella

Since cilia and flagella are complex organelles composed of hundreds of proteins assembled in a very structured manner, the mechanisms underlying their construction have fascinated scientists over years. Deciphering this process could be the key in the understanding of the ciliary diversity and the aetiology of ciliopathies.

**Figure 4:**
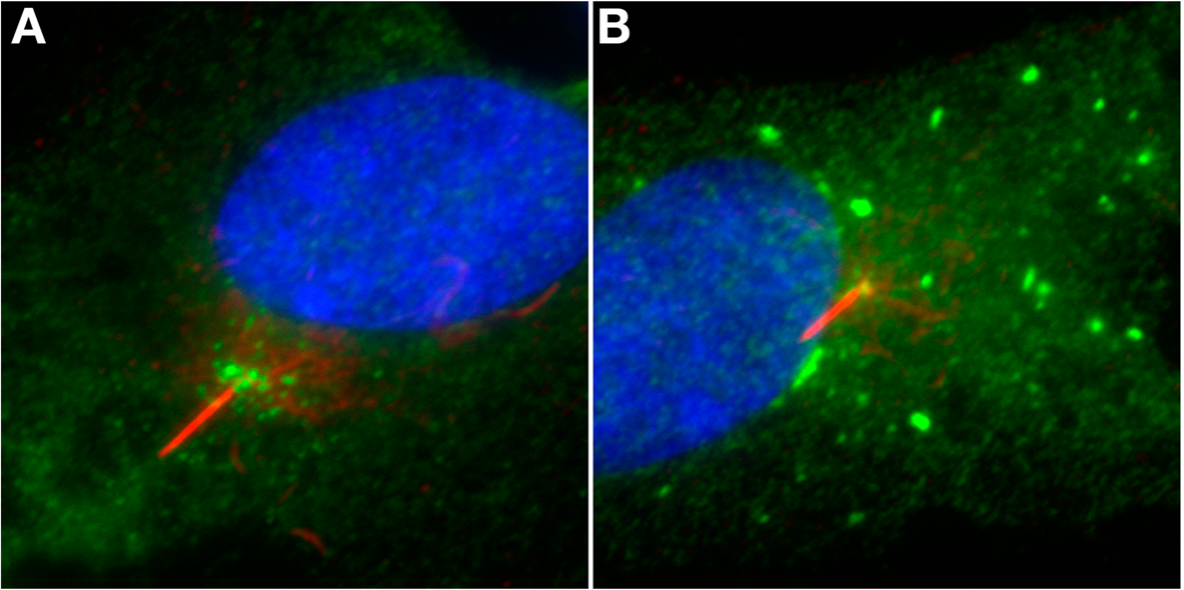
**Example of protein dynamics at the cilium.** A centriolar satellite protein (green) re-localises upon activation of an innate immune response in ciliated (red) RPE-cells. **A**. Unstimulated cell. **B**. Stimulated cell (Credit: Jens Andersen).

Cilia are assembled in the continuity of a basal body that is a modified centriole. Centrioles are the core components of the centrosome, an organelle that plays key functions in many cellular pathways including the organisation of the microtubule network and of the mitotic spindle, signalling, degradation of ubiquitinated proteins through the proteasome and, of course, ciliogenesis [41]. Jens Andersen (University of Southern Denmark, Odense, Denmark) provided evidence that the centrosome as well as centriolar satellites might be involved in innate immune signalling. Indeed, TRIM protein family members, key components of this signalling pathway, are found at the centrosome where they negatively regulate centrosome-dependent signalling (Figure 4). Gillian Griffiths (University of Cambridge, UK) presented the immunological synapse of activated cytotoxic T lymphocyte (CTL) as a ‘frustrated cilium’. Using live imaging, she showed how the centrosome becomes polarized and moves to dock at the plasma membrane where it triggers cytotoxic granule release. While in CTL, the centrosome never grows an axoneme, the intracellular pool of Smo polarises to the immunological synapse, analogous to the Hh-triggered translocation of Smo into the cilium. These data demonstrate that CTL activation triggers Hh signalling, which promotes centrosome polarization, actin remodelling, granule release and target cell killing. These two sets of data point to a key function of the centrosome and of ciliogenesis-associated machineries in the immune response and/or immune cells, despite their lack of cilia and raise questions about the immune status of patients suffering from ciliopathies.

Kenneth Schou (University of Copenhagen, Denmark) identified KIF13B as a new ciliary kinesin. Expression of KIF13B is induced during ciliogenesis: it localises to cilia and acts as a negative regulator of cilium length. It plays an important role in cilium-dependent signalling downstream of IGF1. This signalling function is likely to rely on the ability of KIF13B to interact with the transition zone protein NPHP4, which is required for localization of KIF13B at the basal body and may directly participate in its signalling functions. Pierre Gönczy (École Polytechnique Fédérale de Lausanne, Switzerland) showed that the C2 domain containing centriolar protein SAS-1 is required for centriole stability in *C. elegans* and for formation of the bipolar mitotic spindle in early embryos [42]. Interestingly, SAS-1 has significant homology with C2CD3, a C2 domain containing protein which is required for the elongation/maturation of the distal ends of centrioles and, therefore, for ciliogenesis. *C2CD3* mutations were recently linked to a specific form of oro-facial-digital syndrome [43]. These data raise the possibility that SAS-1 is involved in centriole elongation/maturation in *C. elegans*.

It was recently revealed that actin polymerization negatively regulates ciliogenesis and that ‘relaxing’ the actin cytoskeleton through the use of low doses of cytochalasin D increases cilium length and favours ciliogenesis in non-permissive conditions (presence of serum) or in cell types impaired in their ability to form cilia [44]. Joon Kim (KAIST, Daejeon, Korea) presented data pointing to the role of the Hippo pathway YAP/TAZ transcription factors in this actin-dependent control of ciliogenesis. YAP/TAZ, which are sequestered in the cytoplasm in ciliated cells, operate as negative regulators of ciliogenesis. Kim and collaborators also identified that two kinases, LIMK2 and TESK1, known for their key roles in actin remodelling, act upstream of YAP/TAZ and are therefore also key actors of this actin-dependent control of ciliogenesis. These data are supported by others showing direct ciliary functions/localization of Hippo pathway components [45].

Based on an siRNA screen targeting all kinases (kinome), Gislene Pereira (German Cancer Research Centre, Heidelberg, Germany) showed that MARK4, a centrosome-associated kinase known to negatively regulate MAP family members, is present at the basal body where it controls the initial steps of axoneme elongation [46]. She revealed that MARK4 acts in concert with another kinase, TTBK2, which was shown to play similar functions to MARK4 in ciliogenesis and is implicated in spinocerebellar ataxia. The activity of both kinases is required to fully remove the CEP97/CP110 inhibitory complex from the distal end of the mother centriole allowing axonemal elongation. Interestingly, these two kinases are known to phosphorylate MAPs, including MAP4 that has previously shown to negatively regulate cilium length [47]. Together with data presented by Sophie Saunier (see above), these results suggest that besides their roles in early events in ciliogenesis, these two kinases might have additional regulatory functions on MAPs during axoneme elongation and/or on their functions in cytoplasmic microtubules.

All together, the data presented in this session provided a set of functions for the centrosomes and new clues to better understand how this organelle orchestrates the early steps of ciliogenesis. Next, Daniela Iaconis (Telethon Institute of Genetics and Medicine, Naples, Italy) addressed one of the questions that had evaded molecular understanding: do some proteins get translated near the base of the cilium? Her finding that the translational pre-initiation complex depends on OFD1 for localization as well as specific localization of a subset of mRNA to the base of the cilium marks the first inroad into solving this puzzle.

In almost all organisms, the extension of the axoneme depends on IFT (Additional file 1: Video 1), a bidirectional microtubule-based process whereby large assemblies or ‘trains’ of IFT particles, associated with ciliary cargo such as tubulin, are transported in the anterograde direction along the axoneme by kinesin-2 motors and brought back by cytoplasmic dynein-2. A specific session devoted to IFT revealed new insights into the molecular working of this important process. It was initiated by Pietro Lupetti (University of Siena, Italy) who presented new results from electron tomographic analysis of IFT particle trains in *Chlamydomonas*. Previous work suggested that anterograde IFT trains are long and loosely packed whereas retrograde IFT trains are short and compact [48]. However, new results indicated that anterograde trains could include both long and short subclasses that vary in abundance depending on flagellar length. Cécile Fort (Pasteur Institute, Paris) reported that in *Trypanosoma*, IFT is required only for construction of a new flagellum but is dispensable for the maintenance of flagellum length. This is in contrast to *Chlamydomonas* where IFT is required for flagellar assembly as well as maintenance [49] and could reflect differences in the role of IFT from one cell type to the other [50]. This provides an interesting tool to examine the role of IFT in already assembled flagella. Mary Porter (University of Minnesota, Minneapolis, USA) described the function of the cytoplasmic dynein-2 light intermediate chain (LIC) in *Chlamydomonas* and presented data using two new mutants exhibiting different reduction levels. This suggested that dynein-2 LIC is required for flagellar exit of the BBSome and IFT particles, but not of kinesin-2. Moreover, she revealed that reduction of retrograde transport also impacted on the frequency of anterograde events, supporting previous work in trypanosomes indicating that IFT proteins were cycling between the flagellum and its base [51].

Victor Jensen (Simon Fraser University, Burnaby, Canada) discussed whole organism developmental expression profiling in *C. elegans,* which led to identification of several new ciliary genes, including *RAB28* and *WDR60*, a recently identified cytoplasmic dynein-2 intermediate chain [52]. He further reported that WDR60 and XBX1, a cytoplasmic dynein-2 light chain subunit, travel with separate kinesin-2 motors during anterograde IFT in *C. elegans*, and this may serve to keep the dynein motor inactive. The next speaker, Gerald Liew (Stanford University School of Medicine, USA), showed that IFT27 interacts directly with BBS3/ARL6 and that IFT27 induces ciliary exit of ARL6 and the BBSome, possibly by promoting GDP to GTP exchange in ARL6 at the ciliary tip [53,54]. Esben Lorentzen (Max Planck Institute of Biochemistry, Munich, Germany) presented a detailed interaction map of the entire 15-subunit IFT-B complex, consisting of a 9-subunit so-called core subcomplex [55], as well as an additional subcomplex comprising the remaining 6 IFT-B polypeptides. Previous work had suggested that the latter six polypeptides are peripherally associated with the IFT-B core, but these new data indicate that they form a distinct subcomplex.

What to transport into the cilium and how much of it? For the cilium to grow into and then be maintained as a functional structure, this question is particularly pertinent for the vast quantities of tubulin needed for the ciliary axoneme [56]. Karl Lechtreck (University of Georgia, Athens, GA, USA) presented beautiful work addressing this issue in the green alga *Chlamydomonas* using two-colour live TIRF microscopy simultaneously imaging the bona fide IFT-cargo tubulin and IFT particles (or trains). Observing full-length steady-state cilia, cilia length mutants and cells possessing both growing and non-growing cilia allowed for the analysis of the tubulin (cargo) loading patterns of IFT trains in various ciliary states. This showed that tubulin transport by IFT is upregulated during ciliary growth by a cilium-autonomous mechanism [57].

Defects in the protein CCDC103 cause PCD in zebrafish and humans due to the loss of outer dynein arms [58]. Stephen King and colleagues (University of Connecticut, Farmington, USA) subjected *Chlamydomonas* Ccdc103/Pr46b to ruthless biochemical characterisation and to electron microscopy localization along the entire ciliary axoneme. Ccdc103 turns out to be a very tough protein that can bind directly and very tightly to axonemal microtubules, which it also stabilises. Interestingly, this binding displays a regular spacing of about 12 nm, reminiscent of the 24 nm outer dynein arm repeat pattern. In conjunction with its direct positioning on the microtubule axoneme, Ccdc103 could thus serve as a pattern generator for outer dynein arm assembly of growing motile cilia and flagella. Takashi Hishikawa (Paul Scherrer Institut, Switzerland) presented the organisation of the dynein motor in the flagellum of the green alga *Chlamydomonas*. Using tomography, he revealed the complexity of the inner dynein arms both between microtubule doublets and along a given doublet. The quality of the structural data now allows better modelling of the way dynein molecules interact with microtubules *in situ*, revealing unique specificities of the axonemal dyneins *versus* cytoplasmic dynein [59].

Aberrant dopamine signalling is associated with various forms of epilepsy. Catrina Loucks (Simon Fraser University, Canada) reported that the *C. elegans* ortholog of a human EF-hand gene (*EFHC1*, encoding a protein found in the axoneme [60]), commonly mutated in juvenile myoclonic epilepsy, is expressed specifically in a subset of dopaminergic ciliated sensory neurons and that the protein localises to both cilia and synapses. Mutations in the worm gene plus double mutant analyses with other ciliary and dopamine-related genes generated behavioural phenotypes (related to food availability) that are consistent with increased synaptic dopamine release and signalling. Conceptual and molecular overlaps between sensory cilia and neuronal synapses have previously been noted [61]. It will thus be very interesting to find out what the exact molecular role of this protein is in both cilia and synapses. Of note, the *C. elegans* protein actually lacks the EF-hand domain but is otherwise strongly conserved.

Building on the conceptual and molecular similarities (for example, pore proteins) between gating at the nuclear pore and the ciliary base, Kristen Verhey and her colleagues (University of Michigan, USA) developed a tool to clog the gate by forced dimerization of crucial protein components [62]. This approach makes it possible to examine the characteristics of ciliary entry, as well as how entry and exit might work mechanistically. The author presented a tour-de-force analysis of various cytosolic and membrane proteins slated to enter (or exit) the cilium. Of note, while cytosolic proteins (starting from a certain size) destined for the cilium depend on an active nucleoporin-dependent mechanism (which can be clogged), ciliary membrane proteins do not. Instead, they enter the cilium via a different mechanism. Very soon, this type of approach will yield a detailed model of how ciliary entry and exit work at the molecular level. Julien Santi-Rocca (Pasteur Institute, Paris, France) presented data on the location of RP2 at the base of the transition zone and its function with regard to IFT trafficking in *T. brucei*. He also showed that nucleoporins are present at the base of the flagellum in this organism, indicating an ancient origin for this association.

The meeting ended with the acknowledgements of all sponsors and with a special thanks to Aude Cambon (Institut Pasteur) who was in charge of virtually all the practical aspects of the organisation (website, registration, budget management, gala dinner, abstract book and so on). The award for best student oral presentation (funded by the journal *Mammalian Genome*) was given to Gerald Liew, and those for best student poster presentations (funded by the Société Française de Biologie du Développement and by the GDR CIL) were given to Vera Jansen, Tiffanie Fowlkes-Comninellis, Dylan Bergen, Valentina Grampa and Rita Rua Ferreira. Finally, Ronald Roepman announced that the next conference will be held in Amsterdam from 4th to 7th October 2016.

## Additional file

Additional file 1:
**Video 1.** IFT trafficking in *Trypanosoma brucei.* GFP:IFT52 trafficking in live trypanosomes using laser illumination with spinning disc technology and filmed with an EMCCD camera (credit: [51]).

## Abbreviations

AAV: adeno-associated virus
BBS: Bardet-Biedl syndrome
CTL: cytotoxic lymphocyte
Hh: Hedgehog
ICO: intraciliary oscillation
IFT: intraflagellar transport
LRO: left-right organiser
NPHP: nephronophthisis
PCD: primary ciliary dyskinesia
